# From MDGs experience to SDGs Actions: Insights from Tanzanian Nurses and Midwives

**DOI:** 10.24248/eahrj.v9i2.861

**Published:** 2025-12-24

**Authors:** Peter Taratara, Elaine Bennett, Selma Alliex

**Affiliations:** a Jarrah Patient Assessment and Recovery Centre (PARC), Wodonga, Victoria, Australia; b Perth Campus, Murdoch University, Australia; c The University of Notre Dame, Australia

## Abstract

**Background:**

Nurses and midwives constitute the largest proportion of the health workforce and play a central role in achieving maternal and child health outcomes. During the Millennium Development Goals (MDGs) era, particularly MDGs 4 and 5, their engagement was considered essential. However, anecdotal evidence from low- and middle-income countries suggests that nurses and midwives were insufficiently involved in MDG-related planning and decision-making. This study explored the enabling and inhibiting factors influencing Tanzanian nurses’ and midwives’ participation in the implementation of MDGs 4 and 5, with the aim of identifying lessons to strengthen engagement during the Sustainable Development Goals (SDGs) era.

**Methods:**

A descriptive, holistic case study design was employed. Data were collected in 2017 from five hospitals in Tanzania (three public and two private) using surveys, focus group discussions, and semi-structured interviews. Participants included 66 clinical nurses and midwives and eight nursing and midwifery administrators. Quantitative data were analyzed descriptively, while qualitative data were analyzed using inductive content analysis to identify key themes related to awareness, participation, enablers, inhibitors, and future engagement.

**Results:**

Most participants reported awareness of the MDGs, primarily through mass media, workplace meetings, and colleagues. However, awareness was uneven across facilities, with notable gaps in some hospitals. Nurses’ and midwives’ participation in MDGs 4 and 5 was largely limited to routine clinical activities, such as patient education and record-keeping, rather than involvement in planning or decision-making processes. Major enablers included their professional knowledge, numerical dominance in the workforce, and close proximity to patients. Key barriers included heavy workloads, limited time, inadequate representation in policy forums, insufficient empowerment, and organizational and resource constraints. Despite these challenges, participants expressed strong willingness to engage more actively in future global health initiatives.

**Conclusion:**

Nurses and midwives in Tanzania played a largely indirect and constrained role in the implementation of MDGs 4 and 5, with participation primarily embedded in routine clinical practice rather than strategic decision-making. Strengthening leadership support, improving access to information, enhancing professional empowerment, and increasing involvement in policy and planning processes are critical to maximizing nurses’ and midwives’ contributions to the achievement of Sustainable Development Goal 3 and future global health agendas.

## BACKGROUND

The Millennium Development Goals (MDGs), declared in 2000, provided a global framework consisting of eight priority goals aimed at reducing poverty and improving human development. Among these, MDG 4 sought to reduce child mortality, while MDG 5 aimed to improve maternal health.^4^ The MDGs built upon the earlier “Health for All” agenda rooted in the 1978 Alma-Ata Declaration, which identified primary health care as the cornerstone of equitable health systems.^5^

To operationalize the MDGs, the United Nations (UN) and the World Health Organization (WHO) issued two guiding documents: the UN Global Strategy for Women and Children's Health and WHO's Strategic Directions for Strengthening Nursing and Midwifery Services. Both documents emphasized the need for active engagement of nurses and midwives in achieving MDGs 4 and 5.^6^ These health professionals make up the largest proportion of the global health workforce and occupy frontline roles directly influencing maternal and child health outcomes.^7^ The strategic guidance promoted educational opportunities, improved competencies, better institutional support, interprofessional collaboration and strengthened leadership roles.^8^

Despite these efforts, Tanzania like many Sub-Saharan African countries continued to experience high maternal and child mortality throughout the MDG period.^9^ In 2009, the country ranked 27^th^ globally in under-five mortality, ^10^ with neonatal deaths also significantly above global averages.^11^ Contributing factors included malnutrition, infectious diseases, inadequate living conditions and limited access to essential health services. By 2013, while certain nutritional indicators improved,^12^ maternal mortalities remained high at 410 deaths per 100,000 live births,^13^ and progress toward MDG 5 was off-track.^9^ These challenges were also seen regionally, as Sub-Saharan Africa continued to bear disproportionate maternal mortality burdens.^14^

Anecdotal evidence suggests that nurses and midwives in Tanzania and other developing contexts had limited involvement in MDG-related policy processes and implementation planning. Persistent barriers included inadequate knowledge, low confidence, limited organizational support, few leadership opportunities and persistent professional stereotypes.^15^ Recent global analyses warn that such barriers will impede SDG progress unless health systems strengthen nursing and midwifery leadership and visibility.^2,3^

Nurses and midwives form the backbone of national health care systems and are instrumental in delivering essential health services and advancing key global health agendas.^16^ Understanding the gaps that constrained their participation in MDGs 4 and 5 is essential for improving national health service delivery. Lessons drawn from these constraints will inform strategies that support stronger engagement and more effective implementation of Sustainable Development Goal 3 (good health and well-being).

This research intended to understand these barriers, so that a lesson could be learned that would improve nurses and midwives’ participation during the SDGs phase (2016 – 2030).

## METHODOLOGY

### Study Design, Setting and Participants

This study employed a descriptive, holistic case study design to explore the experiences of nurses and midwives in Tanzania. The descriptive component allowed detailed examination of their perceptions of, and engagement with, the Millennium Development Goals (MDGs), while the holistic case study approach recognized that participation is shaped by contextual, cultural and structural conditions within the health system.^16^ Accordingly, the case encompassed nurses’ and midwives’ awareness of and involvement in the MDGs within broader organizational settings, including educational background, workplace culture and other relevant contextual factors.

Data were collected from five hospitals in Tanzania: two teaching hospitals in Dar es Salaam and three hospitals in Kigoma Region. Of these, three were public facilities; Muhimbili National Hospital, Maweni Regional Hospital and Kasulu District Hospitalv and two were private hospitals, namely Kairuki Hospital (Dar es Salaam) and Kabanga Hospital (Kigoma Region). The study focused on nurses and midwives’ awareness of, and participation in, the MDGs, with particular attention to MDG 4 and MDG 5.

### Recruitment of Participants

Following ethics approval, formal letters were sent to hospital administrators and other relevant authorities inviting them to participate in interviews. Additional letters were sent to the selected hospitals, and posters were displayed on hospital noticeboards inviting clinical staff to volunteer for the survey and focus group (FG) discussions. Nurses and midwives who completed the survey were also invited to join FG sessions.

Participants purposively selected nurses and midwives working in general, pediatric and maternity wards, including those involved in maternal and child health community outreach. Clinical staff working exclusively in operating theatres, intensive care units, emergency departments or general community roles were excluded, as their roles did not involve routine, direct care for mothers and young children.

### Data Collection

Data were collected in 2017, two years after the end of the MDG period and during the second year of the Sustainable Development Goals (SDGs). Phase 1 comprised a survey, focus group discussions and interviews. A total of 74 individuals participated: 66 were clinical nurses and midwives, and eight were administrators with managerial or executive responsibilities within the nursing and midwifery hierarchy. The survey and qualitative instruments captured information on MDG awareness, forms of participation, perceived barriers and facilitators, and perspectives on future engagement in similar global goals. The overall data collection plan is summarized in Table 1.

**TABLE 1:** Data Collection Plan

Data Collection Activities	Attended By	Activity/Location
Data Collection Preparation	The Researcher	Application to UNDA and NIMR to obtain Ethics clearance
Research Tools (Survey/FG/Interviews, Consent Form).	The Researcher	Perth - WA
Session 1	The Administrator	Interview at the administrator's office
Session 2	The Administrator	Interview at the administrator's office
Session 3	Clinical staff (nurses & midwives)	Administration of survey at Muhimbili hospital
Session 4	Clinical staff (nurses and midwives)	Focus group discussion at Muhimbili hospital
Session 5	The Administrator	Interview at the administrator's office
Session 6	The Administrator	Interview at the administrators’ office
Session 7	The Administrator	Interview at the administrator's office
Session 8	Clinical staff (nurses and midwives)	Administration of survey at Kairuki hospital
Session 9	Clinical staff (nurses and midwives)	Focus group session at Kairuki hospital.
Session 10	The Administrator	Interview at administrator's office
Session 11	Clinical staff (nurses and midwives)	Administration of survey at Maweni hospital
Session 12	Clinical staff (nurses and midwives)	Focus group session at Maweni hospital
Session 13	The Administrator	Interview at the administrator's office
Session 14	Clinical staff (nurses and midwives)	Administration of survey at Kasulu hospital
Session 15	Clinical staff (nurses and midwives)	Focus group session at Kasulu hospital
Session 16	Clinical staff (nurses and midwives)	Administration of survey at Kabanga hospital
Session 17	Clinical staff (nurses and midwives)	Focus group session at Kabanga hospital
Session 18	The Administrator	Interview at the administrator's office

The survey was administered in designated hospital meeting rooms. Response rate among attendees was 100%, as each participant returned a completed questionnaire. Immediately after the survey, participants were invited to join an FG session focused on the same domains (awareness, participation, enablers, and inhibitors). Each FG lasted approximately 30–45 minutes and concluded when no new insights were emerging.

Phase 2 involved in-depth interviews with four administrators who had also contributed to Phase 1. This phase was specifically designed to elicit practical strategies for enhancing nurses’ and midwives’ participation in future health-related global goals beyond the MDGs.

### Data Analysis

Closed-ended survey items, including demographic characteristics and other categorical variables, were analyzed using descriptive statistics across all five hospitals. Responses to items such as age, gender, role and years of service were converted into numerical codes and summarized in tables and percentages (for example, Table 2 for age, role and gender). “Yes/No” responses were counted per hospital, and findings related to MDG awareness and participation were presented as frequencies and percentages.

**TABLE 2:** Participants’ Age, Role, Gender

HOSPITAL	PARTICIPANTS’ AGE						ROLE RN/EN		GENDER		HOSPITAL TYPE
	19 – 28	29 – 38	39 – 48	49 – 58	59 – 68	69+	RN	EN	F	M	PUBLIC (PC)/PRIVATE (PV)
MUHIMBILI (23 Participants)	3	10	8	1	1	0	12	11	22	1	PC
KAIRUKI (11 Participants)	1	2	3	5	0	0	5	6	10	1	PV
MAWENI (8 Participants)	1	3	3	1	0	0	2	6	7	1	PC
KASULU (12 Participants)	0	4	6	2	0	0	4	8	8	4	PC
KABANGA (12 participants)	8	2	1	1	0	0	6	6	9	3	PV
TOTAL	13	21	21	10	1	0	29	37	56	10	Total 66

Open-ended survey responses and qualitative data from FGs and interviews in both phases were analyzed using inductive content analysis (ICA), following the steps described by Elo and Kyngäs.^18^ This process comprised data transcription, immersion in the data, development of categories and subcategories aligned with the research questions, formulation of statements and themes, and reporting of findings. Tape-recorded interviews and FGs conducted in Kiswahili were first transcribed verbatim, translated into English, and then developed into interpretive transcripts. Interpretive transcription acknowledges the role of context and subjectivity in meaning-making while maintaining methodological rigor.^19^ All transcripts were checked for accuracy by a second person.

From these data, key categories, concepts and phrases were identified. Particular attention was paid to the research questions. For instance, “MDG awareness” was established as a major analytic area, from which subcategories such as “how made aware”, “why made aware” and “when made aware” were generated. These subcategories guided the search for convergent statements across survey responses, FG discussions and interview data.

### Ethics Approval

Ethical approvals were obtained from the University of Notre Dame Australia Human Research Ethics Office (approval number 016127F), the Tanzania National Institute for Medical Research (NIMR; NIMR/HQ/R.8a/Vol.1X/2334), and Muhimbili National Hospital (MNH; 2016-10-18/AEC/Vol.X1/296).

## RESULTS

### Demographic Characteristics

Sixty-six nurses and midwives from five hospitals completed the survey. Three of these hospitals were public, and two were private. Participants’ ages ranged from 18 to over 60 years. Females comprised 56 participants (85%) and males 10 (15%). Most respondents (n=42, 63.6%) were in the 29 to 38 and 39 to 48-year age groups.

Participants’ years of clinical experience ranged from less than one year to more than 25 years. The largest group (n=22, 33%) reported 6 to 10 years of experience. Seventeen nurses (26%) had 0 to 5 years of experience. The most experienced participants (n=6; 8% of all participants) were based in Dar es Salaam, particularly at Kairuki Hospital. Kabanga Hospital had the highest proportion of nurses with up to five years’ experience (n=9; 14% of all participants).

### Awareness of MDGs – Closed-Ended Responses

Closed-ended survey items showed high levels of reported awareness of the MDGs across most hospitals. In Muhimbili, Kairuki, Maweni and Kabanga, 96%, 82%, 88% and 92% of respondents, respectively, indicated that they had heard about the MDGs. Muhimbili recorded a 96–100% “Yes” response for awareness-related items, while Kairuki reported 82–100% across the three main awareness questions. Maweni's “Yes” responses ranged from 88–100%.

In contrast, Kasulu Hospital recorded only 58% “Yes” to the first awareness question, indicating that 42% of respondents there reported not having heard about the MDGs. For the remaining awareness items, Kasulu and Kabanga registered 100% “Yes” responses. The relatively low awareness at Kasulu suggested a substantial knowledge gap among nurses and midwives in that facility compared with the other hospitals.

### Awareness of MDGs – Open-Ended Responses

Open-ended survey items and qualitative data from FGs (clinical staff) and interviews (administrators) further explored awareness of the MDGs. Participants were asked whether they had heard about the MDGs (Question 1); the target to reduce under-five mortality (U5MR; Question 2); and the target to improve maternal health (Question 3).

Across all hospitals, responses to the first question were predominantly affirmative (90–95%), with the exception of Kasulu, where only 58% of participants responded “Yes”. This again implied that a significant proportion (42%) of nurses and midwives at Kasulu had not heard about the MDGs.

The three most commonly reported sources of information about MDGs were: mass media (television, internet and newspapers), workplace meetings, and information passed on by colleagues. Attendance at dedicated workshops was the least frequently mentioned source.

Administrators reported that their awareness of MDGs was shaped by direct communication, workshops, media exposure and professional associations. For example, one administrator stated:

*“Tanzania is part of the Global health agenda; therefore, we got to know immediately once the MDGs were passed by the United Nations (UN) General Assembly. The Ministry of Health and Social Welfare (MOHSW) informed key stakeholders throughout the country”.* (INT1).

Another explained:

*“We attended some workshops organized by staff from the MOHSW, and during the workshop sessions we got to know about MDGs; …but also, the Nurses and Midwives Association within the hospitals informed their members about MDGs and what needed to be done in the area of child and maternal health; ….and various educators were invited to give information and education about implementation strategies”* (INT 4).

A further administrator noted:

*“A lot of information reached us through media, …but of course, there were letters, memos; and managers attended some workshops organized by Tanzania Midwives’ Association (TAMA); …I believe some nurses and midwives at the bottom of the hierarchy did not hear much about MDGs”* (INT5).

### Participation

In this study, “participation” referred to the extent to which nurses and midwives were involved in decision-making processes and implementation strategies related to MDGs 4 and 5. The following questions were used to examine participation:

- How did Tanzanian nurses and midwives become aware of MDGs?
- How did they participate in MDGs, and who was involved in seminars, workshops and meetings focused on maternal and child health and record-keeping?
- What factors enabled or hindered their participation?
- What lessons from MDG implementation could inform future global health goals?

Survey data indicated that only a minority of nurses and midwives reported participating in workshops organized outside their workplaces. At Muhimbili, 30% of respondents indicated attending such workshops; at Kairuki, 18% reported participation. Maweni recorded 38% participation, while Kasulu and Kabanga reported 29% and 32%, respectively. Across all sites, medical doctors were identified as the main attendees at workshops and seminars, followed by policy-makers and politicians. Nurses and midwives were rarely mentioned among the key participants in high-level seminars or decision-making forums.

Interviewees confirmed that nurses and midwives were actively involved in documenting child and maternal health indicators, especially immunization data. However, survey and FG findings highlighted that their participation in MDGs was largely confined to routine clinical activities rather than active involvement in planning or decision-making. Many participants described their contribution as “embedded in their practice”, and characterized it as “incomplete participation” or even “complete non-participation”.

Clinical staff in the survey and FGs expressed their experiences as follows:

*“I participated by attending normal duties; I gave education to patients in the clinic about different family planning methods”* (SURV4).

*“We provided education on family planning and nutrition to expecting mothers in the clinics, we taught them about the importance of taking vaccines, and this was our usual duties which I believe was part of participation in MDGs”* (SURV7).

*“We did what we normally do every day…we were told there are Millennium goals, but we continued doing what we do at work every day”* (FG2).

*“We were not sufficiently represented in MDGs planning and decisions especially if you think about the numbers of nurses and midwives in health care”* (FG3).

*“The doctors make most decisions. We must learn to voice up our point of views and be able to stand for the interests of our profession”* (SURV1).

### Enablers and Inhibitors

Data from interviews, FGs and surveys identified several factors that supported or inhibited nurses’ and midwives’ participation.

Key enablers included their professional knowledge, close proximity to patients and numerical dominance within the health workforce. These features were described as strengths:

*“Nurses and midwives are the majority in the health care system and are closer to patients than anyone else; the care given to expecting mothers and the young children is very much in the hands of nurses and midwives”* (INTERV2).

*“We are closer to patients, …we participated fully by doing our normal duties; we educated patients in clinics, and attended to all planned training to improve our skills”* (FG4).

*“I have the knowledge and I know what I can do to help. I thought it was a chance for us to work harder to reduce the children mortality. During work meetings the discussions came up, but not much happened”* (SURV10).

Major inhibitors included heavy workloads, lack of time to attend workshops, inadequate representation in decision-making, and resource constraints:

*“We do not get time to be off our normal duties to attend workshops; …we are always overworked, …. we hardly get time to take a break”* (FG5).

*“We were not sufficiently represented in implementation decisions especially if you think about the numbers of nurses and midwives in health care”* (SURV6).

*“Possibly there was not enough funding to get all nurses and midwives mobilized, and I think that the policy makers’ priorities did not target nurses and midwives. Despite all that, we need to demonstrate being confident in ourselves, demonstrate self-drive and support each other”* (SURV 31).

### Future Participation

Nurses and midwives were asked how they would like to participate in future health-care decision-making at facility, regional and national levels. Most expressed a strong desire for greater involvement in planning and implementing future global and national health initiatives. Examples of responses included:

“*The more participation by nurses and midwives, the more the effect will trickle down the health care system and implementation strategies will reach every worker very quickly* (FG1).

*“More future participation means better outcomes in attaining goals”* (SURV 14).

*“Nurses and midwives have the required knowledge, they do more to care for mothers and children; with more participation, there will be better coordinated implementation plans”* (INTERV4).

*“Nurses and midwives are responsible for more than 75% of the work related to child and maternal health care; this is a professional group which needs to be supported at all levels if future goals are to be realized*” (INTERV5).

*“There is need to improve nurses’ and midwives’ training model and leadership skills; the quality of education must focus on skills, competencies, confidence building and ethics”* (INTERV1).

*“Nurses and midwives could constantly share information with doctors and policy makers on how implementation plans are going. Even allied health professionals such would be kept in the loop. …. What I'm saying here is that working together and knowing that each person is a professional in his/her domain is key to success”* (INT4).

In summary, nurses and midwives became aware of MDGs mainly through media, meetings and colleagues. Their participation was largely by implementing decisions made by others, particularly medical doctors. Enablers included their numbers in the workforce, knowledge base and close contact with patients, whereas high workload, professional invisibility and limited funding were major inhibitors. Participants clearly articulated their readiness and desire for more meaningful participation in future health goals.

## DISCUSSION

**FIGURE 1:**
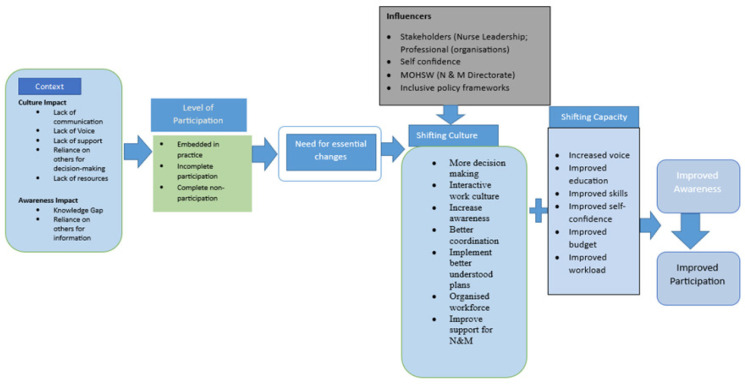
Framework for Future Participation

The discussion is organized around the domains of the Framework for Future Participation (FFP) developed collaboratively with nursing and midwifery leaders and academics: context, level of participation, shifting culture and shifting capacity. The FFP is shown in figure 1.

The United Nations (UN) and the World Health Organization (WHO) explicitly called for nurses and midwives to play active roles in implementing the MDGs, while encouraging each country to design locally relevant strategies. In Tanzania, however, health services during the MDG period were constrained by shortages of human and material resources and by limited collaborative initiatives, fragmented information-sharing and a lack of clear, widely disseminated strategies for implementing MDGs 4 and 5. Similar challenges have been reported in South Africa, where efforts to reduce maternal and child mortality were compromised by insufficient collaboration among health professionals, including traditional healers.^20^

In this study, most nurses and midwives reported learning about the MDGs primarily through mass media, and to a lesser extent through workplace meetings and colleagues. Media messages were generally directed at the public rather than specifically at nurses and midwives, and thus did not provide detailed, profession-specific guidance on implementation. These findings align with studies that describe persistent barriers to implementing evidence-based maternal health interventions in low-and middle-income countries, including Tanzania, where poor collaboration, weak communication and limited information-sharing remain significant obstacles.^21^

Lomazzi et al. similarly reported that limited awareness and inadequate dissemination of information among health professionals in some African countries contributed to the non-attainment of MDGs, emphasizing the importance of targeted publicity and engagement of key players.^22^ Awareness, understood as being conscious of information, context and one's role in planned activities is critical for participation, accountability and effective implementation.^23^

Information and knowledge sharing in organizations involve the exchange of routine, work-related messages and reports that underpin planned activities and strategic goals.^24^ Such sharing enhances staff insight, “know-how” and alignment with organizational objectives. It is closely related to the Sense of Coherence (SOC) framework, which highlights comprehensibility (ability to understand information), manageability (capacity to use available resources) and meaningfulness (commitment to engage with tasks) as key components of workplace engagement.^25^ Limited awareness and poor information-sharing therefore represent substantial barriers to achieving global health goals.

### Level of pParticipation and Professional Visibility

Nurses’ and midwives’ participation in MDG implementation in this study was largely partial or indirect. They implemented decisions made elsewhere but had limited influence on the design of strategies or policies. Many described their involvement as “incomplete participation”, where they carried out clinical tasks derived from higher-level decisions. “Complete non-participation” referred to their exclusion from decision-making at district, regional and national levels.

Motivational theories suggest that people are more likely to commit to and work towards goals when they understand which behaviors are required, feel competent to perform those behaviors, and view the goals as meaningful.^26^ When nurses and midwives are excluded from planning and decision-making, their motivation and sense of ownership over implementation may be diminished.

These findings contrast with the expectations articulated in the International Council of Nurses’ Leadership for Change (LFC) program, which emphasizes nurses’ leadership in addressing national health challenges and calls on them to be actively involved in health policy formulation.^26^ LFC advocates for nurses and midwives to assume responsibility for quality care and maximize their contribution to health system performance.

In many low- and middle-income countries, nurses and midwives deliver a large proportion—sometimes up to 90%—of primary health care, often at equal or lower cost than physicians.^27^ They are recognized as central to health service delivery and to achieving global initiatives such as the MDGs and SDGs.^28^ Yet, they frequently remain underrepresented in strategic decision-making and under-recognized as key actors in health policy discussions.^29^

In this Tanzanian case study, nurses’ and midwives’ participation in MDGs was predominantly embedded in their everyday clinical work, while decision-making forums were dominated by medical doctors, policy-makers and politicians. This illustrates an ongoing gap between the rhetorical recognition of nurses and midwives as central to global health agendas and their actual involvement in planning and governance.

### Shifting culture and shifting capacity

A growing body of literature describes the structural and cultural challenges nurses and midwives face in their workplaces, including oppression and marginalization associated with medical dominance and media representations, ^30,31^ staff shortages and high workloads, ^30,32^ resource constraints, lack of support, ^32,33^ and limited voice in organizational decisions.^33^ Many of these challenges were evident in the Tanzanian context, where participants reported heavy workloads, insufficient resources and equipment, inadequate support, and weak cooperation between nurses, midwives and doctors.

Addressing these challenges requires attention from nursing and midwifery leadership at multiple levels. The framework for future participation developed in this study proposes two interlinked domains shifting culture and shifting capacity through which change can be pursued.

### Shifting culture

Workplace and organizational cultures are largely shaped by leadership practices that define “how things are done here”, including patterns of social interaction, cohesion and support.^30^ Effective leadership can create positive, supportive environments that promote collaboration across professional groups, enhance critical thinking and encourage accountability. At the micro level, constructive organizational cultures have been linked to more personcentered and ethically grounded care, which is particularly important for maternal and child health.^34^

Conversely, cultures characterized by rigid hierarchies, limited support and organizational apathy can undermine quality performance and impede effective clinical practice.^35^ Within the shifting culture domain, nursing and midwifery leaders are called to foster mechanisms that improve access to information, strengthen support systems, and create conditions that enable nurses and midwives to participate meaningfully in key decisions.

### Shifting capacity

Shifting capacity refers to strengthening nurses’ and midwives’ voice, knowledge, skills and resource base. It is closely associated with interprofessional cooperation (IPC), interprofessional education (IPE) and critical social theory (CST). IPC entails collaborative initiatives among health professionals who share responsibility for patient care and coordinate tasks across disciplinary boundaries.^36^ IPE brings different professional groups together to learn with, from and about each other with the aim of improving service delivery and patient outcomes.^37^ In several countries, including the United Kingdom, IPE has been used as a strategic tool for driving health policy change and promoting more collaborative practice.^38^

IPC and IPE are particularly relevant in addressing the entrenched power imbalances that often privilege physicians over other health professionals and can strain interprofessional relationships.^36^ Over time, such imbalances have shaped the professional identity of nurses and midwives and their relationship with the medical profession.^39^

CST further contributes to the shifting capacity domain by highlighting three cognitive orientations in knowledge acquisition: technical, practical and emancipatory interests.^40^ Technical interests relate to scientific knowledge; practical interests concern communication and interaction; and emancipatory interests involve critical self-awareness, recognition of constraints and development of strategies to overcome them. For nurses and midwives, university education and ongoing professional development not only build clinical competence but also strengthen confidence, political awareness and capacity for advocacy.

To enhance future participation in global health goals, nursing and midwifery education in Tanzania needs to extend beyond clinical skills to address socio-political, structural and contextual issues that influence practice and decision-making. Communication and relational skills are crucial not only in direct patient care but also in engaging with the broader determinants of nursing and midwifery work.

Nursing and midwifery leaders are therefore urged to take a proactive role in understanding the context of practice, shaping and transforming workplace culture, and creating empowering structures and support systems within the health-care system. The framework's vision of shifting capacity will be realized only if it is underpinned by strong leadership and informed by IPC, IPE and CST principles.

### Study Limitations

The first limitation was that the data collection methods used in this study (survey, interviews, and focus group discussions) were conducted in Kiswahili which is the national language of Tanzania. Although the participants who contributed to the data collection understood English and Kiswahili very well, there was potential risk that some words could lose meaning during translation and data transcription.

The next limitation was that the results for this study were based on a single case study, and the data were collected from five hospitals in two regions of Tanzania. The findings from this single case study may therefore not be generalizable for all hospitals, regions, and districts in the whole of Tanzania.

Yet another limitation was that the researcher had no access to such information such as staff attendance to meetings, workshops or correspondences related to MDGs planning or implementation. The researcher was informed that the information was unavailable. This information would enrich the study.

## CONCLUSION

This study examined how Tanzanian nurses and midwives drew on their experiences with MDGs to inform participation in the current SDG era. The primary aim was to identify factors that enabled or constrained their involvement in implementing MDGs 4 and 5 and to derive lessons for future participation.

Findings indicated that nurses and midwives had limited awareness of and involvement in MDG planning and implementation. The contexts in which they worked were characterized by weak communication, limited professional collaboration and inadequate information-sharing about strategies to meet MDG targets. Health facilities faced shortages of human and material resources, and nurses and midwives experienced limited mobilization, support and representation in decision-making. Their participation was primarily embedded in routine clinical duties and was often described as incomplete or as non-participation.

The discussion integrated empirical findings with existing literature and a framework for future participation, focusing on awareness, participation and readiness for future engagement. Challenges included contextual constraints, workplace culture, the dominance of the medical model and historically unequal relationships between nurses, midwives and physicians. For nurses and midwives to fully realize their potential contribution to achieving future national and global health goals, strong and committed nursing and midwifery leadership is required to empower staff, improve their visibility, and navigate complex work environments through promoting constructive change.

### Recommendations

Understanding the strengths and weaknesses inherent in nursing and midwifery practice is essential for planning future contributions to health goals such as the SDGs. Based on the findings of this study, several recommendations are proposed: 1. Strengthen professional empowerment Nurses and midwives in Tanzania would benefit from an enabling environment that supports advanced education (including postgraduate study) and continuing professional development (CPD). Such conditions would help enhance clinical competence, assertive communication and professional voice; 2. Integrate SDGs into nursing and midwifery education Nurse and midwifery educators are well positioned to improve the visibility and participation of the profession in SDGs. Undergraduate and postgraduate curricula should explicitly include SDGs and related targets, along with the potential roles of nurses and midwives in implementation; 3. Promote leadership development and advocacy skills Leadership training should focus on building confidence, ethical practice, advocacy and policy engagement so that nurses and midwives can contribute effectively to decision-making at all levels of the health system; 4. Enhance interprofessional collaboration and information-sharing Mechanisms for regular communication and joint planning between nurses, midwives, doctors and other health professionals should be strengthened to ensure coordinated and inclusive approaches to maternal and child health goals.
